# Depressive Symptoms Among South African Construction Workers: Associations with Demographic, Social and Work-Related Factors, and Substance Use †

**DOI:** 10.3390/ijerph22050694

**Published:** 2025-04-27

**Authors:** Rita Peihua Zhang, Paul Bowen, Peter Edwards

**Affiliations:** 1 School of Property, Construction and Project Management, RMIT University, GPO Box 2476, Melbourne, VIC 3001, Australia; peter.edwards@rmit.edu.au; 2 Department of Construction Economics and Management, University of Cape Town, Private Bag X3, Rondebosch, Cape Town 7701, South Africa; paul.bowen@uct.ac.za

**Keywords:** depressive symptoms, determinants, male construction workers, South Africa

## Abstract

The construction industry exhibits higher rates of depression in its workforce compared to other industries. This study investigates the association between the prevalence of depressive symptoms and various demographic (e.g., age, ethnicity, education), social, and work-related factors (e.g., relationship status, living environment, work situation) and behavioural factors (e.g., alcohol and drug use). Survey data collected from 496 male construction workers working in the Western Cape were analysed using binomial logistic regression to determine the associations. The results showed that ‘Black African’ construction workers exhibited lower levels of depressive symptoms than ‘Other’ ethnic groups, and workers with at least secondary education had the highest levels of depressive symptoms compared to workers with other levels of education. Workers who were single or living with other adults without children had a significantly higher risk of depression compared to those in other forms of family relationships. Substance use was found to be associated with lower levels of depressive symptoms, suggesting that construction workers use alcohol and drugs as a coping strategy for short-term depressive symptom reduction. Construction organisations should develop strategies to protect the mental health of construction workers, particularly those who are prone to depression.

## 1. Introduction

Depression is a global mental health concern, significantly contributing to the overall burden of disease. The Global Burden of Disease (GBD) 2019 survey revealed that mental disorders accounted for 4.9% of worldwide disability-adjusted life years (DALY), with depressive disorders accounting for 37.3% of this category [1]. The WHO [2] states that *“DALYs for a disease or health condition are the sum of years of life lost due to premature mortality (YLLs) and years of healthy life lost due to disability (YLDs) due to prevalent cases of the disease or health condition in a population*”. The impact of depression extends across various facets of an individual’s life, from work productivity to social relationships and community engagement [3,4]. The effects of depression can be long-lasting or recurrent, hampering an individual’s functioning and overall quality of life [4].

The construction industry stands out as a high-risk sector for mental illness [5,6], and individuals employed in construction face a heightened risk of depression compared to those in other industries [7]. This risk is particularly pronounced among manual and trade construction workers [8]. Depression is a significant cause of suicide [3], and this warrants attention in the construction industry where workers experience significantly higher suicide rates compared to the general work population. In England, between 2011 and 2015, the suicide rate among skilled construction and building trade workers was 1.6 times higher than the national average, and the risk of suicide among low-skilled construction workers was 3.7 times higher [9]. In Australia, male construction workers are twice as likely to commit suicide than non-construction workers [10].

Previous studies have shown a strong link between poor mental health experienced by construction workers and the construction work environment, which is characterised by high job demand (e.g., long work hours, high workload), low job control, low job security, workplace injustice (e.g., discrimination, harassment, and bullying), and poor workplace social support [5,8,11]. While these studies have provided essential insights into how work contributes to construction workers’ mental disorders and what workplace changes are needed to protect workers’ mental health, they are limited in identifying individual or demographic characteristics that render specific groups within the construction industry more susceptible to mental disorders. In addition, most of mental health studies in the construction industry have concentrated on developed countries, whereas the prevalence and causes of mental disorders in developing countries, such as South Africa, have not been sufficiently examined [12].

**Figure 1:**
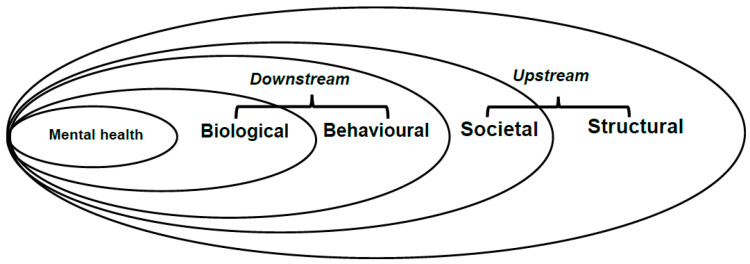
Conceptual mental health model (Source: Western Cape Department of Health and Wellness [13]).

The health and disease model put forth by the Western Cape Department of Health and Wellness in South Africa proposes that individual health is influenced by a spectrum of factors, including biological (e.g., age and ethnicity), behavioural (e.g., substance use), societal (e.g., living and working conditions), and structural factors (e.g., socioeconomic status). The model has been adapted and is shown in Figure 1.

Aligned with this adapted model, our study investigates the associations between the prevalence of depressive symptoms and various demographic factors (age, ethnicity, and education), social- and work-related factors (relationship status, living arrangement, and work status), and behavioural factors (alcohol and drug use). The study also contributes to the existing body of knowledge by focusing on the developing country of South Africa. It is expected that our study would draw attention to the prevalence and nature of depressive symptoms in the construction industry in South Africa, through identifying factors that contribute towards this mental disorder and suggesting strategies by which it may be addressed.

## 2. Literature Review and Hypothesis Development

### 2.1. Depression and Depressive Symptoms

Depression is characterised by feelings of sadness, loss of interest, guilt, and low self-esteem, and individuals with depression often experience sleep and appetite disturbances, tiredness, difficulty in concentration, and suicidal thoughts [4]. Depressive disorders encompass two significant sub-categories: (a) major depressive disorder or depressive episode; and (b) dysthymia or persistent depressive disorder. The former sub-category includes symptoms, such as depressed mood, loss of interest, and low energy. The severity of a depressive episode can range from mild to moderate or severe, contingent on the number and intensity of symptoms. The latter sub-category presents as chronic, mild depression. The symptoms may resemble those of a depressive episode, but they tend to be less intense and more enduring [14].

Depression can manifest as a recurrent or chronic condition, often resulting in a significant impairment of a person’s ability to fulfil their professional, familial, and societal roles [3]. This global burden of depression is substantial, impacting 3.8% of the world’s population, with 5.0% of adults and 5.7% of older individuals (aged 60 and above) bearing the brunt [4]. Alarmingly, the African region, with South Africa in particular, reports some of the highest prevalence rates of depressive disorders among all the World Health Organization (WHO) regions [14]. Additionally, studies indicate that the prevalence of depression is significantly more pronounced among construction workers compared to the general working population [5,15]. This points to the likelihood of a heightened prevalence of depressive symptoms among construction workers in South Africa.

In the context of the construction industry, mental health issues, such as depression, exert a profound influence on both productivity and safety [16]. The construction environment is inherently hazardous, and requires workers to have sufficient mental capacities to remain concentrated and alert and to manage various safety hazards on site [5]. These capacities can be impaired when construction workers experience depression, leading to higher risks of workplace accidents and injuries. Indeed, studies indicate a higher incidence of work-related injuries among workers with depression [17]. Thus, there is the need to examine what factors potentially contribute to construction workers’ experience of depression and to suggest preventive strategies to protect their mental health.

### 2.2. Age, Ethnicity, and Education

The prevalence rates of depression vary by age in the general population, peaking in older adulthood (aged 55–74 years) with a rate above 7.5% among females and above 5.5% among males [14]. In the construction industry, there is also a trend that older workers experience a higher level of depression prevalence compared to younger workers. For example, Park and Jeong [18] reported that the prevalence of depressive symptoms increased with age among South Korean construction workers. Specifically, the rate of depressive symptoms experienced in the age group of 60 and above (41.9%) was significantly higher than the rates in the age groups of 50’s (37.1%) and those under 50 (32.2%). This trend may be related to age-related changes in workers. People experience natural physical and cognitive changes when they age, including declining physiological functions, reduced physical strength and endurance, changes in hearing and vision, slower information processing speed, and reduced ability to respond quickly and effectively to stressful events [19,20]. These changes have clear implications for workers’ health and well-being in the construction work environment, which is physically and psychologically demanding. De Zwart et al. [21] reported that, compared to younger construction workers, older workers had significantly more complaints about the work being physically demanding, working under time pressure, low job security, and unfavourable changes in the work environment. Age-related changes and their associated impacts on work experience subsequently contributes to workers’ deteriorating health, including mental health [20]. We can therefore hypothesise that:

**H1.**  *Older construction workers are more likely to present with more depressive symptoms compared to younger construction workers in South Africa.*

Ethnicity has emerged as a significant factor in the risk of mental health issues, and studies have shown that individuals from ethnically disadvantaged groups are more susceptible to mental illness [22,23]. These groups often face racial discrimination, have a lower socioeconomic status, and encounter stressful life events, all of which contribute to an increased risk of depression [22]. Hamad et al. [24] conducted a study among low-income South African adults and found that individuals from “non-white” racial backgrounds, including black, coloured, or Indian populations, were associated with higher levels of depressive symptoms. In the US, research suggests that ethnically disadvantaged groups, such as African and Spanish-American communities, are less likely to seek help, have reduced access to mental health services, and are less likely to use psychotropic medication compared to white individuals. These groups often experience more prolonged, chronic, and severe forms of depression [23].

Studies have also explored the health experiences of ethnically disadvantaged migrant construction workers. For instance, Ang et al. [25] investigated a group of migrant workers in Singapore, a majority of whom were employed in the construction industry. Their findings revealed that Bangladeshi workers were at a higher risk of psychological distress, largely due to financial barriers that hindered their access to effective healthcare. Similarly, Palaniappan et al. [26] reported that Bangladeshi construction workers in Singapore had a high prevalence of depression. Specifically, workers who reported being ill and requiring regular medication were more likely to experience depression compared to those who did not report such health issues. This indicates that construction workers from ethnically disadvantaged backgrounds are more likely to face financial difficulties and encounter obstacles in accessing health resources and services, resulting in a higher risk of mental illness. Building on these insights, it is hypothesised that:

**H2.**  *In the South African construction industry, “black” African workers are more likely to exhibit a higher prevalence of depressive symptoms compared to individuals from “other” ethnic backgrounds.*

Education is a socioeconomic status indicator that plays a pivotal role in influencing an individual’s risk of experiencing depression. Research by Freeman et al. [27] in three European countries, showed that an increase in the number of years of education tends to decrease the likelihood of depression across these countries. Education is a personal resource that has far-reaching effects on other socioeconomic status indicators, such as employment, occupation, and earnings, all of which are protective factors for mental health [28]. Moreover, education is a form of ‘human capital’ that generates a range of other resources, assisting individuals in more effectively pursuing their fundamental needs, including emotional well-being [28]. However, in the context of the construction industry, Palaniappan et al. [26] reported a counterintuitive finding. Their study suggested that construction workers with higher levels of education might experience higher levels of depressive symptoms than those with lower levels of education. This unexpected result could be related to the nature of construction work, some of which is manually intensive and does not necessarily require a high level of formal education or skill. More highly educated construction workers may find that the tasks they are performing do not align with the level of education or training they have received, potentially leading to a heightened risk of dissatisfaction and, subsequently, depression [26]. This accords with previous research that indicates that skill underutilisation is a significant risk factor for depressive symptoms among male workers in manufacturing settings [29]. Taking these research findings into consideration, the following hypothesis is formulated:

**H3.**  *In the South African construction industry, workers with higher levels of education are more likely to exhibit a higher prevalence of depressive symptoms compared to their less educated counterparts.*

### 2.3. Relationship Status, Living Arrangements and Work Status

Marital status is a social factor that can influence an individual’s experience of depression. Research indicates that married individuals tend to report lower levels of depressive symptoms compared to those who are separated, divorced, widowed, or never married [30,31]. Furthermore, LaPierre [31] observed that marital status contributes to variations in individuals’ experiences of depressive symptoms, and these differences tend to become more pronounced over time. LaPierre [31] suggested that this phenomenon can be explained by two theories: the marital resource theory and the cumulative advantage/disadvantage theory. The first theory suggests that marital status partially determines an individual’s access to resources and exposure to stressors, leading people with different marital statuses to have varying experiences related to depression [31,32]. The second theory posits that inequalities between groups can increase over time based on initial advantages or disadvantages, thereby predicting greater divergences in depressive symptoms according to marital status [31,33].

Marriage has also been identified as a protective factor for mental health among workers in the construction industry. Dong et al. [34] conducted a study on American construction workers and reported that individuals who were not married were 1.7 times more likely to experience serious psychological problems and 1.8 times more likely to experience suicidal ideation compared to their married counterparts. Based on these findings, a hypothesis can be formulated:

**H4.**  *Single workers in the South African construction industry are more likely to exhibit a higher prevalence of depressive symptoms compared to workers in married or long-term relationships.*

However, the context of South Africa introduces a unique aspect to marital status. In South Africa, being married does not necessarily imply living with one’s family, which can be attributed to the political history of the country. During the apartheid era, single-person households were deliberately created by the government to discourage the urbanisation of black African families ([35]; cited in [36]). Black Africans, mainly males, who migrated from rural areas to cities for work were often accommodated in single-living arrangements, ensuring that their families remained in their rural homes. Even after the apartheid legislation was removed, living alone continued to increase in the post-apartheid period [36]. This is because individual migrant labour patterns persisted due to a lack of affordable family accommodation in urban areas [36], coupled with substantial growth in informal squatter settlements with poor living conditions that were not suitable for families [37,38]. Much of the research on the impact of living alone on mental health, although often focusing on older people, has identified a strong link between living alone and an increased risk of depression among elders [39,40]. The risk of depression increases because people who live alone are more likely to experience social isolation and receive less emotional and instrumental support compared to people who live with others [40]. It is reasonable to extend this concern to individuals of younger age groups beyond just older adults. Building on this context and prior research, an additional hypothesis can be proposed:

**H5.**  *Workers in the South African construction industry who live alone are more likely to exhibit a higher prevalence of depressive symptoms compared to workers who cohabit with others.*

Work status and employment arrangements play a crucial role in shaping the mental health of workers. A notable trend in the global labour market is the substantial growth of precarious employment, including short-term contracts, casual jobs, and part-time work [41]. Precarious employment is characterised by uncertainty, instability, and insecurity, and individuals in such positions often bear all the risks associated with work (as opposed to employers or the government bearing at least some of these risks), with limited access to social benefits and statutory entitlements [42]. Precarious employment is strongly associated with perceptions of job insecurity, which, in turn, can have a detrimental impact on workers’ overall health [43]. Research has shown that among various employment arrangements, casual full-time workers tend to experience the poorest psychosocial work conditions. These conditions include a lack of job control, night shift work, high exposure to job hazards, high job strain, and high job insecurity, all of which contribute to poor mental health [44].

In the context of the construction industry, Dong et al. [34] reported that American construction workers employed on a part-time basis were more likely to experience a higher prevalence of mental illness and suicidal ideation compared to those employed on a full-time basis. Highly casualised employment structures are prevalent in the South African construction industry, with full-time permanent employment being relatively rare, except among highly skilled artisans. In light of this context, the following hypothesis is formulated:

**H6.**  *Construction workers on casual or temporary contracts in the South African construction industry are more likely to exhibit a higher prevalence of depressive symptoms compared to their counterparts in permanent positions.*

### 2.4. Alcohol Consumption, Drug Use/Abuse, and Depressive Symptoms

The literature highlights a reciprocal association between substance use and depression. On one hand, individuals often turn to substances, such as alcohol and drugs, as a coping mechanism to alleviate mental tension or strain, a concept known as the tension-reduction theory [45]. Consistent with this theory, a survey conducted by Drinkaware [46] among a sample of adults in the UK population revealed that 38.0% of respondents reported drinking to forget about problems, 47.0% to change their bad mood, and 41.0% to reduce feelings of depression or nervousness. In the construction industry, research also suggests that construction workers use substances as a coping strategy to deal with work-induced stress, anxiety, and depression [47,48,49]. On the other hand, substance use further promotes or exacerbates the problems of depression and anxiety, leading to poorer mental health [50]. Alcohol, for example, is known as a central nervous system depressant. A study among UK adults found that, among individuals who use alcohol as a form of self-medication to cope with issues, those who are diagnosed as harmful and probable dependence drinkers are more likely to experience low mental well-being compared to those who are diagnosed as low risk and hazardous drinkers [51].

In the construction industry, Dong et al. [34] reported that construction workers who had used illicit opioids and other types of illegal drugs in the past year, as well as those who had reported alcohol dependence or abuse in the past year, were more likely to experience poor mental health and have suicidal thoughts compared to non-users. Taking this evidence into consideration, two hypotheses are proposed:

**H7.**  *In the South African construction industry, workers who score as moderate to high risk for alcohol-related harm on validated tests are more likely to exhibit a higher prevalence of depressive symptoms compared to workers who score as low risk for alcohol-related harm.*

**H8.**  *In the South African construction industry, workers who score as at possible risk of drug-related problems or heavily dependent on drugs on validated tests are more likely to exhibit a higher prevalence of depressive symptoms compared to workers who score as having no drug-related problems.*

## 3. The Conceptual Research Model

**Figure 2:**
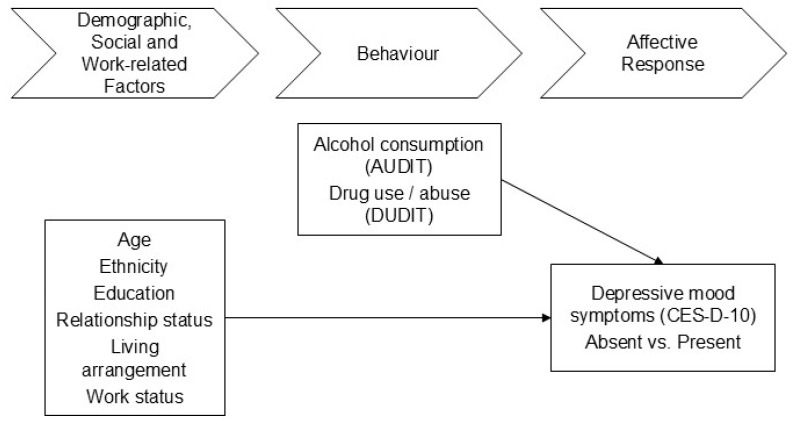
Graphical overview of the research model (Source: Own processing).

A conceptual research model of factors predicting depressive symptoms among South African male construction workers is proposed (see Figure 2). The model is the framework for developing and testing individual hypotheses. It assumes that depressive symptoms are predicted by a range of demographic, social, and work-related factors, including age, ethnicity, education, relationship status, living arrangements, and work status. The model also conjects that depressive symptoms are determined by behavioural/lifestyle factors, including alcohol assumption and drug abuse.

## 4. Research Method

The epistemological and methodological assumptions underlying this study are positivist in nature. This means that the study is grounded in the belief that empirical, observable, and measurable data can be used to gain knowledge and understanding. Additionally, the ontological paradigm employed is objectivist–determinist, which assumes that depressive symptoms among South African construction workers may be either partially or fully determined by the demographic, social, and work-related factors, as well as the behavioural and lifestyle choices they make.

### 4.1. Research Design

For data collection, a survey instrument was utilised as the primary method. It was administered on construction sites and encompassed three standardised tests to assess depressive symptoms, alcohol use, and drug use. It also included questions aimed at gathering demographic, social, and work-related information. The survey design was based on an instrument originally developed by the Human Sciences Research Council (HSRC) of South Africa.

### 4.2. Instrument and Measures

Table 1 provides an overview of the demographic, social, work-related, and behavioural variables, along with the response options for each.

The level of depressive symptoms among the workers was assessed using the 10-item CES-D-10 scale (US Center for Epidemiologic Studies Depression Scale) [52] as presented in Table S1 in the Supplementary File. The Cronbach’s alpha value for CES-D-10 was 0.90, indicating strong internal consistency.

To evaluate alcohol consumption, the 10-item Alcohol Use Disorders Identification Test (AUDIT) [53] was employed, with details outlined in Table S2 in the Supplementary File. The Cronbach’s alpha value for AUDIT was 0.89, suggesting good scale reliability.

For assessing workers’ drug use and potential abuse, the 11-item Drug Use Disorders Identification Test (DUDIT) [54] was used, and relevant information is provided in Table S3 in the Supplementary File. The Cronbach’s alpha value for DUDIT was 0.91, indicating a high internal consistency.

All three tests are well-validated scales, with higher scores on each indicating higher levels of the construct of interest.

### 4.3. Participants and Setting

The study population was drawn from 18 construction sites affiliated with 7 companies in the Western Cape. The selection of these construction companies, sites, and participants was based on a purposive and convenient sampling technique [55]. The sample included all male workers present on the construction site during the field researchers’ visit. This diverse group of on-site workers encompassed both unskilled and skilled labourers, as well as office staff, such as wage clerks and technicians. Note that our research is restricted to male workers as we could not find sufficient female construction workers to constitute a reliable sample.

To ensure that language was not a barrier to participation, the questionnaire was translated into three of South Africa’s 11 official languages: English, Afrikaans, and IsiXhosa. These languages were selected based on their prevalence in the Western Cape. Experienced bilingual translators, recruited with the assistance of the Human Sciences Research Council of South Africa (HSRC), were responsible for the translations. Field researchers, who were fluent in all three languages, were present at each site to assist in clarifying the meaning of a question if a participant had difficulty understanding it in their chosen language version. The field researchers did not provide answers or prompt responses. Prior to conducting each survey, permission was obtained from each of the participating construction companies, and the research received ethical approval from a South African university. To ensure the reliability of the questionnaire translated into different languages, the internal consistency of each of the three scales (CES-D-10, AUDIT, and DUDIT) was checked for each ethnic group. The results showed little differences in Cronbach’s alpha values, indicating that participants across groups had a good understanding of the questions.

Participants completed the questionnaires in a designated office container facility equipped with chairs and a table. Wherever possible, care was taken to ensure that participants had sufficient privacy to complete the questionnaire without being disturbed by others. Where the number of people required it, the survey was conducted in a relay format at several sites. Before participating, potential participants were informed about the survey and assured that it was voluntary, anonymous, and confidential. After obtaining informed consent, participants proceeded to complete the questionnaire. The amount of time taken to complete the questionnaire varied depending on the educational level and literacy of the participants.

## 5. Data Analysis

### 5.1. Data Cleaning

The initial dataset consisted of 576 returned questionnaires. Two of these were disregarded as they were found to be blank. An additional 18 responses were excluded because they did not provide answers to key questions related to alcohol and drug use. Among the remaining 556 responses, 60 were removed due to an excessive amount of missing values (i.e., exceeding 15.0%) following the criteria established by Graham [56]. The final dataset used for analysis thus comprised 496 valid responses. To address the missing data, the EM (expectation maximisation) algorithm was employed for imputation.

### 5.2. Analysis Method

The dataset was initially screened for any anomalies. Discussion of the psychometric validation of the factorial structure of the CES-D-10, AUDIT, and DUDIT scales and an examination of the construct validity (convergent and divergent) of the three scales are not provided here as the psychometric validation process has previously been established (see [52,57,58,59]).

For the CES-D-10, AUDIT, and DUDIT scores, categorical cut-off scores were applied to categorise the scale responses. The CES-D-10 scores were divided into two groups: those below the clinical cut-off level of 10 and those equal to or above 10, indicating the absence and presence of depressive symptoms, respectively. This choice aligns with the suggestions of Andresen et al. [52]. The AUDIT scores were categorised into four groups: ‘0–7’ indicating low risk of harm, ‘8–15’ for medium risk, ‘16–19’ for high risk or harmful level, and ‘20 or more’ for dependence likely. The DUDIT scores fell into three categories: ‘0–5’ suggesting no drug-related problems, ‘6–24’ for possible drug-related problems, and ‘25 and above’ signifying probably highly drug-dependent individuals.

Descriptive analysis was utilised to summarise demographic, social, and work-related data. The frequencies and percentages reported relate to the number of valid responses to individual questions. Participants’ age, ethnicity, education level, relationship status, living arrangement, and employment status were categorised as shown in Table 1. Data analysis was performed using SPSS V28.0 [60].

Bivariate tests (χ2 test or Fisher’s Exact Test) were used to examine associations between depressive symptoms (absence and presence), categorical variables (demographic, social and work-related information), and the two scales of interest (AUDIT and DUDIT). Binomial logistic regression was then used to assess the strength of the relationship between the predictor variables (demographic, social and work-related, and behavioural) and the binary dependent variable representing depressive symptoms in construction workers. Predictive modelling of this nature is best performed using logistic regression, which can adequately handle non-contiguous categorical data, as it focuses on factors that are likely to lead to workers not having or having a depressive symptom profile. Multiple regression is not suitable for this situation. Other analysis techniques such as ANOVA are also inappropriate in this context as they deal with variables that are continuous. Logistic regression is suitable for testing models that predict categorical outcomes with more than one category—as in this study (absence versus presence of depressive symptoms). Predictors (independent variables) can take different forms, for example, they can be categorical, continuous or a mixture of both—as in this study.

## 6. Results

### 6.1. Participant Characteristics

The characteristics of the all-male participants in the final data set are shown in Table 2. Ages ranged between 18 and 67 years (M = 35, Md = 34), with the majority in the 26–35 age group, indicating a relatively young participant group. Regarding ethnicity, approximately 59.0% of the participants were classified as ‘black Africans,’ using the ethnic categories that were previously employed to distinguish historically disadvantaged groups in South Africa. To maintain numerical balance for the analysis, all other participants were re-categorised as ‘other’ in terms of ethnicity. In terms of educational attainment, nearly 20% of the participants had at most primary education, while the majority (65.5%) had received secondary education or had exposure to it. Nearly half (48.2%) were married or in a long-term relationship. In terms of employment status, 53.1% were in casual or short-term contract employment.

Ninety participants (18.1%) scored at least 10 on the CES-D-10 depression scale, indicating depressive symptoms. For alcohol consumption, 24.9% (*n* = 123) showed at least moderate risk of alcohol harm (score 8 or above) on the AUDIT test whilst 7.4% (*n* = 37) showed high risk to possible dependence (score 16 or greater). For the DUDIT test, 5.9% (*n* = 29) of the study participants showed the possible presence of a drug problem, and 0.8% (*n* = 4) showed high levels of drug dependence.

### 6.2. Bivariate Relationships Between Depressive Symptoms and Characteristics of Participants

Bivariate tests of association were employed to examine the relationship between the level of depressive symptoms (CES-D-10 score: absence or presence) and each of the participant characteristics (i.e., demographic, social and work-related, and behavioural).

A significant association was indicated between depressive symptoms and ethnicity, living arrangements, levels of alcohol consumption, and extent of drug use but not with age, education (on the cusp, *p* = 0.052), relationship status, or work status (see Table 2).

### 6.3. Binomial Logistic Regression Analysis

To further explore the determinants of depressive symptoms experienced by construction workers, a binomial logistic regression was conducted using participants’ demographic, social- and work-related, and behavioural characteristics (see Table 3).

Table 3 shows the association between the different categories of predictor variables and the depression profile, in contrast to the reference category. The probability (*p*-value) indicates the significance of each category of the predictor variable relative to the reference category. The odds ratio (OR) indicates the effect size.

From Table 3, the odds ratio for ‘other’ ethnicities was greater than 1. The statistic indicates that this ethnic grouping was 1.9 times more likely than ‘black African’ workers to be classified as having more depressive symptoms compared to being classified as having an absence of such symptoms.

Workers having attained or having been exposed to secondary school education were 2.99 times more likely to present with depressive symptoms than were workers who had experienced at most primary school education. There was no difference in the presence of depressive symptoms between workers with primary or less education and those having obtained or been exposed to a tertiary education.

The odds ratio for workers either married or in a long-term relationship was less than 1 (0.41). This indicates that married and long-term relationship employees are less likely than single employees to present with depressive symptoms. Married workers and workers in long-term relationships experienced a reduction of 59.0% (The following formula can be used to quantify the change in the odds: Change in Odds %: |(OR − 1)| × 100, which is expressed as 0.41 − 1 = |−0.59| × 100 = 59) in the odds of presenting with depressive symptoms compared to single workers.

Regarding living arrangements, workers living with other adults without the presence of children were 12.11 times more likely to present with depressive symptoms than were workers who lived alone. There was no difference in the presence of depressive symptoms between workers living with adults and children or with children only and those living alone.

With regard to the alcohol harm risk profile of workers, the odds ratios for both moderate and high harm risk were 0.20 and 0.23, respectively. This indicates that workers at moderate or high harm risk are less likely to present with depressive symptoms than those at low harm risk. Workers at moderate or high risk of harm were 80.0% and 77.0% less likely to present depressive symptoms, respectively. There was no difference in the presence of depressive symptoms between workers exhibiting likely alcohol dependency and those at low risk of harm.

Regarding drug use and the presence of depressive symptoms, the odds ratio for both the possible presence of drug-related problems, and a high level of drug dependency, was 0.03. This indicates that workers experiencing either possible drug-related problems or a high level of drug dependency are less likely than workers with an absence of drug-related problems to present with depressive symptoms.

No association was found between age and the absence or presence of depressive symptoms. Additionally, there are no significant differences observed between the categories of work status and the depressive symptoms classification (see Table 3).

Logistic regression analysis does not assume a distribution of predictor scores but is sensitive to high levels of correlation among predictors. Preliminary analyses did not uncover any significant issues related to high predictor correlations. For the categorical predictors, the study employed dummy variables to contrast different categories, such as various ethnic groups and education levels. A reference category was selected for each predictor variable, and all other categories were compared to this reference category. The chosen reference categories were ethnicity (‘black African’), education (primary education and below), relationship status (single), living arrangements (living alone), and work status (casual or contract). The logistic regression analysis aimed to determine whether specific demographic, social, work-related, and behavioural characteristics made construction workers more or less likely to exhibit depressive symptoms. The results revealed that the predictors, when considered together as a set, reliably distinguished between workers who exhibited depressive symptoms and those who did not (χ2 = 18.813, *p* < 0.05, df = 8). The −2 log-likelihood was 401.234, and the model demonstrated a good fit, as confirmed by the Hosmer and Lemeshow goodness-of-fit test (χ2 = 7.227, *p* = 0.512 with df = 8). The overall prediction success rate was 83.0%.

Table 4 provides a summary of the hypothesis testing results, offering insights into how the various factors contribute to the presence of depressive symptoms among construction workers.

## 7. Discussion

The following discussion derives from the binomial regression analysis of the relationship between depressive symptoms and the demographic, social and work-related, and behavioural characteristics of participants.

### 7.1. Associations Between Demographic Factors and Presence of Depressive Symptoms

The findings indicated that younger workers were just as likely to experience depressive symptoms as their older counterparts. This observation may be linked to the challenging socioeconomic situation in South Africa, where the unemployment rate had been steadily increasing, reaching a peak of 35.3% at the end of 2021 [61]. Notably, the highest unemployment rates were among young people, with 66.5% for those aged 15–24 and 43.5% for those aged 25–34. These high unemployment rates, coupled with the associated job insecurity, could contribute to younger construction workers’ feelings of depression.

Interestingly, the hypothesis that ‘black African’ workers would be more likely to experience depressive symptoms compared to ‘other’ ethnicities was not supported by the findings. Other studies have suggested that the association between ethnicity and depression can be affected by the nature of measures used for assessing depressive symptoms. For instance, Riolo et al. [23] report that the major depressive disorder (MDD) has a significantly higher prevalence in whites than in African and Mexican Americans, while the opposite pattern is noticed for dysthymic disorder. Similarly, David et al. [62] found that the life MDD prevalences were higher for whites than for Caribbean blacks and African Americans; however, the chronicity of MDD was higher for black groups than for whites. This is because blacks with MDD do not often seek or receive treatment, thus experiencing more chronic and prolonged depression. In the present study, the CES-D-10 scale was intended to assess symptoms relating to major depressive disorder, not dysthymic disorder, which could explain why the present study found that ‘black African’ construction workers experience fewer depressive symptoms than ‘other’ ethnicities. Another possible reason is the residual effects of the earlier government regime of apartheid [63], also known as the ‘ongoing traumatic stress’ of apartheid [64]. Jackson et al. [65] found that, in South Africa, coloured people reported significantly more anger and hostility (a clear indicator of psychological distress) than all other racial groups. The political history of Coloured people in South Africa could explain their higher levels of anger/hostility. More specifically, the majority of Coloured voters supported the conservative National Party in the 1994 elections, despite having previously been previously disenfranchised by the National Party during its time of government. Coloured people may be torn between their desire for change that improves the lives of South Africans and the expectation that their continuing support for the National Party would give them access to more resources [65]. In our sample, the ‘other’ group was predominantly made up of coloured participants.

The hypothesis that South African construction workers with higher levels of education experience more depressive symptoms compared to less educated workers is partially supported. The results showed that only workers who completed or were exposed to secondary education showed a significantly higher likelihood of presenting with more depressive symptoms than workers with primary education or less. Educational attainment is still low in South Africa. According to OECD [66], among the adult group of 25–34 years old, only 15% achieved a tertiary education, 39% attained an upper secondary education, and 46% achieved a below upper secondary education. It can be assumed that construction workers with a secondary (particularly upper secondary) education can be considered as relatively well educated in South Africa. Consistent with Palaniappan et al. [26], construction workers with a secondary education may perceive that manual construction work and its associated payment levels are not commensurate with their education and skill levels, which contributes to their feeling of depression. However, those construction workers with tertiary education (within the South African construction industry context, this is more related to vocational training and diploma degrees) are likely to be engaged in more managerial roles (e.g., site agent, wages clerk, quantity surveying technician) and thus associated with better remuneration and work conditions, which are protective factors to mental health [28].

### 7.2. Associations Between Social and Work-Related Factors and Presence of Depressive Symptoms

Aligned with our expectations, the research findings demonstrate that married or long-term partnered construction workers experience lower levels of depressive symptoms compared to their single counterparts. This result supports the marital resource model, which suggests that marital status provides individuals with essential resources, such as social and emotional support, that can act as protective factors for mental health [31,32].

Interestingly, our research shows that construction workers who live with other adults, without children, experience significantly higher risk of depression compared to construction workers who live alone. This result appears to be contrary to previous research findings that living alone contributes to the risk of depression due to social isolation [39,40]. This result may need to be interpreted in the South African context. As mentioned earlier, the prevailing migrant labour system combined with inadequate housing for migrant workers in South Africa has led many construction workers to live separately from their families and live in informal settlements [36]. Construction workers who reported living with other adults without children were most likely sharing accommodation with other male migrant workers. Informal settlements for migrant workers are often in poor condition with a lack of basic facilities and amenities [38]. Sharing accommodation in such conditions is likely to add to crowdedness and conflicts, heightening the risk of depression for construction workers [67].

Our study revealed that work status is not a factor that differentiates between construction workers with high and low levels of depressive symptoms in South Africa. As mentioned earlier, South Africa has been facing the social-economic challenges of high unemployment rates. With such a labour market, workers who still have a job (regardless of the employment type) may be psychologically protected due to the positive effects of just being employed. Indeed, Borra and Gómez-García [68] reported the positive effect of ‘others unemployment’ on the wellbeing of employed workers in Spain during a period of economic recession and high unemployment.

### 7.3. Association Between Substance Use and Presence of Depressive Symptoms

Contrary to expectations, we found that higher levels of substance use were associated with lower levels of depressive symptoms among the participants. This result aligns with a study by Langdon and Sawang [49], which also suggested that substance use is commonly adopted as a coping mechanism by construction workers for short-term relief from mental distress. However, it is important to note that this relationship between substance use and reduced depressive symptoms does not necessarily imply a positive outcome. Research by Kushner et al. [69] suggests that substance use and mental disorders can develop into co-morbidity through a vicious feed-forward cycle. In this cycle, individuals may initially use substances to cope with mental distress and experience a short-term reduction in symptoms but then become increasingly tempted to continue using substances, which can further impair their mental health. This, in turn, promotes more substance use, thus forming a cycle of dependence. Therefore, relying on substance use as a short-term strategy for reducing depression symptoms may lead to long-term negative impacts on the health and well-being of construction workers.

## 8. Conclusions and Recommendations

We investigated the associations between the prevalence of depressive disorders and various demographic factors, social and work-related factors, and behavioural factors among site-based male construction workers in the South African construction industry in the Western Cape. The research found that ‘black African’ workers experience lower levels of depressive mood symptoms compared to ‘other’ ethnicities, while workers with secondary education experience higher levels of depressive symptoms compared to workers with other education levels.

Our findings support a call for the adoption of more nuanced diagnostic tools that consider the assessment of chronicity of depressive disorder. They also suggest that it is important for construction organisations to consider the fit between the type of work undertaken and the levels of knowledge and skills possessed by workers, avoiding the issue of underutilisation of skills. Construction workers who live with other adults without children experience significantly higher risk of depression compared to construction workers who live alone. This can be attributed to the migrant labour system and its association with the reliance on housing in informal settlements in the context of South Africa. Sharing accommodation with other migrant males in informal settlements may add to the levels of stress and elevate the risk of depression. These results highlight the need for the provincial and national governments, and construction organisations, to provide adequate housing to migrant construction workers in order to protect their mental health. Additionally, construction organisations should implement initiatives to increase construction workers’ awareness of the long-term harm that substance use may cause to their health and wellbeing. Carefully prepared ‘tool-box’ meetings may be an appropriate way of improving such awareness, but effective communication will be a key aspect to such meetings, given the diversity of ethnicity, languages and literacy. In its “duty of care” (fiduciary responsibility), the construction industry should encourage employer organisations to mitigate the adverse effects of factors that impact upon the mental well-being of workers.

### Limitations and Future Research

Our study is subject to several limitations. First, the sampling strategy employed was purposive, so the findings cannot be generalised to the entire population of South African male construction workers. Despite this, the findings can be regarded as indicative. Future studies using random sampling techniques are recommended to validate the findings.

A second limitation is that only *male* construction workers were included in the sample and did not consider the participants’ self-identified gender identity. Specifically, the survey instrument did not include items that distinguish between the participant’s assigned sex at birth and their self-identified gender identity, a key principle of the neoliberal self-image, which considers each person to be the ultimate authority over their gender identity [70]. In most cases, when field researchers arrived at the site, the site manager had already prepared a group of male workers to be briefed about the study. Although only male workers were purposively sampled, it is not certain if these participants truly self-identified as male. Therefore, it is not possible to make any inferences about how depressive symptoms might differ according to the sexual identity of the participating workers. Future studies should include items assessing workers’ self-identified gender identity. Another limitation is the self-reporting nature of this study through the use of questionnaires. Future study can be conducted to determine drug use through biochemical methods, and compare results obtained by self-reported survey and clinical assessments.

Finally, although logistic regression was used, the analytical process is not standardised, and the outputs remain subject to researcher bias. Future research incorporating gender self-identification, differences in sexuality, new approaches to validating self-reports, and interpretive and qualitative methods would provide validity and further insight into the associations identified in this study.

## Figures and Tables

**Table 1:** Demographic, social, and work-related characteristics of participants (Source: Own processing).

Characteristic	Response Options and Scoring
Age	Years
Ethnicity	‘Black’ African = 1; ‘Other’ = 2
Education	Primary or less = 1; Secondary exposed or completed = 2; Tertiary exposed or completed = 3
Relationship status	Divorced, separated, widowed, or never married = 0; Married or living with a partner = 1
Living arrangements	‘Live alone = 1’; ‘Live with other adults; no children’ = 2; ‘Live with other adults and children < 18 yrs. = 3’; ‘Live only with children < 18 yrs. = 4’
Work status	Casual or contract = 1; Permanent = 2

**Table 2:** Bivariate tests of association between depressive symptoms and demographic, social and work-related, and behavioural characteristics of participants (*n* = 496) (Source: Own processing).

Characteristics	Total	%	Absence of Depressive Symptoms		Presence of Depressive Symptoms		χ^2^ *p*
			*n*	%	*n*	%	
***Depressive symptoms***	496	100	406	81.9	90	18.1	−
(Absence vs. presence)							
***Demographic, social and work-related characteristics***							
Age (years)	-	-	-	-	-	-	0.454 **^+^**
Race/ethnicity							0.024 **^§^**
*‘Black’ African*	293	59.3	230	56.9	63	70.0	
*‘Others’*	201	40.7	174	43.1	27	30.0	
Education (exposed or completed)							0.052
*Primary*	86	18.2	63	16.2	23	27.4	
*Secondary*	309	65.5	259	66.8	50	59.5	
*Tertiary*	77	16.3	66	17.0	11	13.1	
Relationship status							0.342 **^§^**
*Single*	248	51.8	208	52.8	40	47.1	
*Married / Long-term relationship*	231	48.2	186	47.2	45	52.9	
Living arrangements							0.025 **^§^**
*Live alone*	90	19.2	66	17.0	24	29.6	
*Live with other adults; no children*	90	19.2	72	18.5	18	22.2	
*Live with other adults and children < 18 yrs.*	257	54.8	221	57.0	36	44.5	
*Live only with children < 18 yrs.*	32	6.8	29	7.5	3	3.7	
Work status							0.190 **^§^**
*Casual or contract*	254	53.1	214	54.6	40	46.5	
*Permanent*	224	46.9	178	45.4	46	53.5	
***Behavioural characteristics***							
AUDIT score (alcohol consumption)							0.009 **^§^**
*Low risk of harm*	371	75.1	308	76.2	63	70.0	
*Moderate risk of harm*	86	17.5	72	17.8	14	15.6	
*High risk of harm*	18	3.6	14	3.5	4	4.4	
*Likely dependence*	19	3.8	10	2.5	9	10.0	
DUDIT score (drug use/abuse)							0.012 **^§^**
*Absence of drug-related problems*	465	94.1	383	94.8	82	91.1	
*Possible drug-related problems*	25	5.1	20	5.0	5	5.6	
*High level of drug dependency*	4	0.8	1	0.2	3	3.3	

Notes: **^§^** Fisher’s exact test used rather than chi-square test for independence. **^+^** Independent samples *t*-Test.

**Table 3:** Binomial logistic regression model for the relationship between depressive symptoms and demographic, social and work-related, and behavioural characteristics of participants (*n* = 496) (Source: Own processing).

	Adjusted Odds Ratios (aOR) ^+^	
	aOR	95% CI
***Demographic characteristics***		
Age (years)	0.97	0.94–1.00
Race/ethnicity		
*‘Black’ African*	-	-
*‘Others’*	1.90 *	1.04–3.47
Education (exposed or completed)		
*Primary*	-	-
*Secondary*	2.99 *	1.21–7.41
*Tertiary*	1.26	0.57–2.79
***Social and work-related characteristics***		
Relationship status		
*Single*	-	-
*Married/Long-term relationship*	0.41 **	0.21–0.81
Living arrangements		
*Live alone*	-	-
*Live with other adults; no children*	12.11 *	1.47–99.96
*Live with other adults and children < 18 yrs.*	7.25	0.88–59.65
*Live only with children < 18 yrs.*	3.92	0.50–30.93
Work status		
*Casual or contract*	-	-
*Permanent*	0.65	0.37–1.14
***Behavioural characteristics***		
AUDIT score (alcohol consumption) (AC)		
*Low risk of harm*	-	-
*Moderate risk of harm*	0.20 **	0.06–0.60
*High risk of harm*	0.23 *	0.07–0.78
*Likely dependence*	0.25	0.04–1.60
DUDIT score (drug use/abuse) (DU)		
*Absence of drug-related problems*	-	-
*Possible drug-related problems*	0.03 **	0.003–0.04
*High level of drug dependency*	0.03 *	0.002–0.51

^+^ Model adjusted for all covariates; * *p*< 0.05; ** *p*< 0.01.

**Table 4:** Summary of hypothesis testing (Source: Own processing).

Hypotheses	Results
**H1:** Older construction workers are more likely to present with more depressive symptoms compared to younger construction workers in South Africa	Not supported
**H2:** In the South African construction industry, “Black” African workers are more likely to exhibit a higher prevalence of depressive symptoms compared to individuals from “Other” ethnic backgrounds	Not supported
**H3:** In the South African construction industry, workers with higher levels of education are more likely to exhibit a higher prevalence of depressive symptoms compared to their less educated counterparts	Partially supported
**H5:** Workers in the South African construction industry who live alone are more likely to exhibit a higher prevalence of depressive symptoms compared to workers who cohabit with others	Not supported
**H6:** Construction workers on casual or temporary contracts in the South African construction industry are more likely to exhibit a higher prevalence of depressive symptoms compared to their counterparts in permanent positions	Not supported
**H8**: In the South African construction industry, workers who score as at possible risk of drug-related problems or heavily dependent on drugs on validated tests are more likely to exhibit a higher prevalence of depressive symptoms compared to workers who score as having no drug-related problems	Not supported

## Data Availability

The datasets presented in this article are not readily available because of the sensitive nature of the research and the associated ethical restrictions. Requests to access the datasets should be directed to Paul Bowen.

## Supplementary Materials

The following supporting information can be downloaded at: https://www.mdpi.com/article/10.3390/ijerph22050694/s1, Table S1: Items for the CES-D-10 Depressive symptoms scale; Table S2: Scale Items for the AUDIT; Table S3: Scale Items for the DUDIT.
