# Supplementary material for: Depressive Symptoms Among South African Construction Workers: Associations with Demographic, Social and Work-Related Factors, and Substance Use
Source: Int J Environ Res Public Health. 2025 Apr 27;22(5):694. doi: 10.3390/ijerph22050694 (PMC12110885; doi:10.3390/ijerph22050694)
Supplement: Supplementary file 1 [file ijerph-22-00694-s001.zip › ijerph-3480192-supplementary.pdf]

Sumpplementary materials

Table S1. Items for the CES-D-10 Depressive symptoms scale [52].

Items	Response Options and Scoring
During the last week	
DMS1. I was bothered by things that usually don't bother me.	0 = Rarely or none of the time (less than 1 day)<br>1 = Some or a little of the time (1-2 days)<br>2 = Occasionally or moderate amount of the time (3-4 days)<br>3 = All of the time (5-7 days)
DMS2. I had trouble keeping my mind on what I was doing.	As per Item 1
DMS3. I felt depressed.	As per Item 1
DMS4. I felt that everything I did was an effort.	As per Item 1
DMS5. I felt hopeless about the future.	As per Item 1
DMS6. I felt fearful.	As per Item 1
DMS7. My sleep was restless.	As per Item 1
DMS8.I was unhappy.	As per Item 1
DMS9. I felt lonely.	As per Item 1
DMS10. I could not "get going".	As per Item 1

Note: Score range of CES-D-10: 0–30. Presence of depressed mood symptoms - score cut-off $\geq$ 10.

Table S2. Scale Items for the AUDIT [53].

Construct and Items	Response Options and Scoring
**(a) Alcohol Consumption**	
1. How often do you have a drink containing alcohol?	0 = Never (Skip to the end)<br>1 = Monthly or less<br>2 = 2-4 times a month<br>3 = 2-3 times a week<br>4 = 4 or more times a week
2. How many drinks containing alcohol do you have on a typical day when you are drinking?	0 = 1 or 2<br>1 = 3 or 4<br>2 = 5 or 6<br>3 = 7, 8, or 9<br>4 = 10 or more
3. How often do you have six or more drinks on one occasion?	0 = Never<br>1 = Less than monthly<br>2 = Monthly<br>3 = Weekly<br>4 = Daily or almost daily
**(b) Alcohol Dependence**	
4. How often during the last year have you found that you were not able to stop drinking once you had started?	As per item 3
5. How often during the last year have you failed to do what was normally expected from you because of drinking?	As per Item 3
6. How often during the last year have you needed a first drink in the morning to get yourself going after a heavy drinking session?	As per Item 3
**(c) Harmful Alcohol Use**	
7. How often during the last year have you had a feeling of guilt or remorse after drinking?	As per Item 3
8. How often during the last year have you been unable to remember what happened the night before because you had been drinking?	As per Item 3
9. Have you or someone else been injured as a result of your drinking?	0 = No<br>2 = Yes, but not in the last year<br>4 = Yes, during the last year
10. Has a relative or friend or doctor or another health worker been concerned about your drinking or suggested you cut down?	0 = No<br>2 = Yes, but not in the last year<br>4 = Yes, during the last year

Notes: Score range of AUDIT: 0 – 40. Scoring the level of risk: 0-7 (low risk of harm); 8-15 (moderate risk of harm); 16-19 (high-risk or harmful level); and 20 or more (dependence likely).

Table S3. Scale Items for the DUDIT [54].

Items	Response Options and Scoring
1. How often do you use drugs other than alcohol?	0 = Never (Skip to the end)<br>1 = Once a month or less often<br>2 = 2-4 times a month<br>3 = 2-3 times a week<br>4 = 4 times a week or more often
2. Do you use more than one type of drug on the same occasion?	0 = Never<br>1 = Once a month or less often<br>2 = 2-4 times a month<br>3 = 2-3 times a week<br>4 = 4 times a week or more often
3. How many times do you take drugs on a typical day when you use drugs?	0 = 0<br>1 = 1-2<br>2 = 3-4<br>3 = 5-6<br>4 = 7 or more
4. How often are you influenced heavily by drugs?	0 = Never<br>1 = Less often than once a month<br>2 = Every month<br>3 = Every week<br>4 = Daily or almost every day
5. Over the past year, have you felt that your longing for drugs was so strong that you could not resist it?	As per Item 4
6. Has it happened, over the past year, that you have not been able to stop taking drugs once you started?	As per Item 4
7. How often over the past year have you taken drugs and then neglected to do something you should have done?	As per Item 4
8. How often over the past year have you needed to take a drug the morning after heavy drug use the day before?	As per Item 4
9. How often over the past year have you had guilt feelings or a bad conscience because you used drugs?	As per Item 4
10. Have you or anyone else been hurt (mentally or physically) because you used drugs?	0 = No<br>2 = Yes, but not over the past year<br>4 = Yes, over the past year
11. Has a relative or a friend, a doctor or a nurse, or anyone else, been worried about your drug use or suggested to you that you should stop using drugs?	0 = No<br>2 = Yes, but not over the past year<br>4 = Yes, over the past year

Notes: Score range of DUDIT: 0 – 44. Scoring the level of risk: (a) Males: 0-5 (no drug-related problems); 6-24 (possible drug-related problems); 25 or above (probably heavily dependent on drugs); Females: 0-1 (no drug-related problems); 2-24 (possible drug-related problems); 25 or above (probably heavily dependent on drugs).
